# Current and Future Perspectives on the Structural Identification of Small Molecules in Biological Systems

**DOI:** 10.3390/metabo6040046

**Published:** 2016-12-15

**Authors:** Daniel A. Dias, Oliver A.H. Jones, David J. Beale, Berin A. Boughton, Devin Benheim, Konstantinos A. Kouremenos, Jean-Luc Wolfender, David S. Wishart

**Affiliations:** 1 School of Health and Biomedical Sciences, RMIT University, 3083 Melbourne, Australia; 2 Australian Centre for Research on Separation Science (ACROSS), School of Science, RMIT University, 3001 Melbourne, Australia; oliver.jones@rmit.edu.au; 3 Commonwealth Scientific and Industrial Research Organisation (CSIRO), Land and Water, EcoSciences Precinct, 4001 Brisbane, Australia; David.Beale@csiro.au; 4 Metabolomics Australia, School of Biosciences, The University of Melbourne, 3010 Parkville, Australia; baboug@unimelb.edu.au; 5 Department of Physiology, Anatomy and Microbiology, Latrobe University, 3086 Melbourne, Australia; D.Benheim@latrobe.edu.au; 6 Metabolomics Australia, Bio21 Molecular Science and Biotechnology Institute, The University of Melbourne, 3010 Parkville, Australia; konstantinos.kouremenos@unimelb.edu.au; 7 Phytochemistry and Bioactive Natural Products, School of Pharmaceutical Sciences, University of Geneva, University of Lausanne, CMU Rue Michel Servet 1, 1211 Geneva 11, Switzerland; Jean-Luc.Wolfender@unige.ch; 8 Departments of Computing Science and Biological Sciences, University of Alberta, Edmonton, T6G 2E8 AB, Canada; dwishart@ualberta.ca

**Keywords:** structure elucidation, metabolomics, nuclear magnetic resonance spectroscopy, mass spectrometry, Fourier transform-infrared spectroscopy, metabolite profiling

## Abstract

Although significant advances have been made in recent years, the structural elucidation of small molecules continues to remain a challenging issue for metabolite profiling. Many metabolomic studies feature unknown compounds; sometimes even in the list of features identified as “statistically significant” in the study. Such metabolic “dark matter” means that much of the potential information collected by metabolomics studies is lost. Accurate structure elucidation allows researchers to identify these compounds. This in turn, facilitates downstream metabolite pathway analysis, and a better understanding of the underlying biology of the system under investigation. This review covers a range of methods for the structural elucidation of individual compounds, including those based on gas and liquid chromatography hyphenated to mass spectrometry, single and multi-dimensional nuclear magnetic resonance spectroscopy, and high-resolution mass spectrometry and includes discussion of data standardization. Future perspectives in structure elucidation are also discussed; with a focus on the potential development of instruments and techniques, in both nuclear magnetic resonance spectroscopy and mass spectrometry that, may help solve some of the current issues that are hampering the complete identification of metabolite structure and function.

## 1. Introduction

### 1.1. Structure Elucidation

Elucidating the structure of small molecules (typically <1500 *m*/*z*), such as primary and secondary metabolites, is a challenging task for even the experienced spectroscopist. Nevertheless, there are a number of fields in which the “absolute” structural identification of organic compounds is critical; these include natural product chemistry [1,2], clinical biochemistry [3,4,5], drug assessment [6], and forensic science [7]. Classically, structural elucidation of a single metabolite or a class of isolated secondary metabolites (e.g., natural products), is solved by first principles and, is governed to a large extent by the sensitivity (or lack thereof) of nuclear magnetic resonance spectroscopy (NMR) [8] and mass spectrometry (MS) techniques (both are discussed in more detail below). It is important to note that structure elucidation in natural product research (and more broadly in metabolomics) requires detailed information relating to the assay and analytical platform(s) utilized, reference libraries used, and any post analysis screening and results. This typically includes UV spectra, melting and boiling points and any related physiochemical information/properties. However, providing in-depth commentary on these data requirements is outside the scope of this review. As such, the interested reader is directed to the position paper by Inglese et al. on data reporting requirements in high through screening of small molecules [9].

One of the first major issues to be overcome in the structural identification of any small organic compound(s) is the ability to isolate compounds in high purity and in reasonable quantities for downstream analyses (i.e., structure elucidation, biological screening, toxicological studies etc.) as many studies rely on multiple chromatographic purification steps to increase yield and purity.

Another common issue is the stability, or more likely, instability of the isolated compound(s). Factors which may cause compound degradation during and/or after isolation may be due to sensitivity to light, oxidation, other chemicals and/or temperature. Thus, the precipitous acquisition of all spectroscopic data (preferably all at one time) will generally assist in the characterization of the target compound of interest. The assembly of the absolute identification is further supported by the addition of other analytical techniques such as mass spectrometry, Fourier-Transform InfraRed (FTIR) spectroscopy, ultraviolet-visible (UV-Vis) absorption spectroscopy, polarimetry, circular dichroism, X-ray crystallography and so forth. Such methods provide evidence for the functionalities and validation of the proposed structure. Today, the field of metabolomics exploits several of these analytical platforms (i.e., GC/LC-MS and NMR) to simultaneously identify hundreds of metabolites present in a biological system combined with multivariate statistical and computational analyses but these methods are not so well utilized for structure identification.

### 1.2. Structure Elucidation by Nuclear Magnetic Resonance Spectroscopy (NMR)

For an isolated compound, structure elucidation usually begins with NMR (the theory and application of which are discussed below) and the amount of the isolated compound typically in the high ng–mg range. By using routine two-dimensional (2D) NMR experiments (e.g., HSQC (Heteronuclear Single Quantum Coherence), COSY (COrrelation SpectroscopY), TOCSY (Total Correlation SpectroscopY, HMBC (Heteronuclear Multiple Bond Correlation), NOESY (Nuclear Overhauser effect spectroscopy and ROESY (Rotating frame nuclear Overhauser effect spectroscopy) experiments) the potential, absolute structure of an individual compound can be elucidated [10]. The last 10 years has seen the development of a large number of 2D NMR pulse sequences to assist in the elucidation of organic molecules *in situ*. For example, Gierth et al. [11] designed two complementary 2D experiments, 2D PANSY-gCOSY (PArallel NMR SpectroscopY)—Correlation Spectroscopy) and 2D PANSY-TOCSY (Parallel NMR SpectroscopY)—Total Correlation Spectroscopy), which in combination often provide sufficient information for the complete structure elucidation of the majority of small organic molecules in solution.

There has been a concomitant increase in research towards creating NMR chemical-shift prediction tools to increase the rapid identification of small molecules. Such examples includes the commercially available software, ACD Lab’s Structure Elucidator (which includes ^1^H, ^13^C, ^15^N, ^19^F, ^31^P NMR prediction), and is used to dereplicate natural product extracts [12]. Structure Elucidator is regarded as one of the most advanced structure predictive tools available. SENECA is a freeware program for Computer Assisted Structure Elucidation (CASE), that allows for the determination of the correct structure and causes of human errors to be detected. This can be very helpful to the spectroscopist, especially when (NMR) spectroscopic data can lead to more than one structural assignment [13]. Alternatively, CAST (CAnonical-representation of STereochemistry)/CNMR Structure Elucidator is a system which uses sophisticated algorithms to elucidate exactly a correct structure. If not all fragments are present in the database the program proposes relevant substructures that can assist the spectroscopist to identify the actual chemical structures from the available data [14]. There are also a number of open-source structure elucidations related packages such as Logic for Structure Elucidation (LSD) which is used to determine all possible molecular structures of an organic compound that are compatible with its spectroscopic data [15,16] and Automics, a NMR metabolomics NMR alignment with integrated statistics [17]. Additionally, there are a number of open-source databases such as the Madison Metabolomics Consortium Database (MMCD), consisting of experimental data collected under standard conditions, literature data, chemical shifts from theoretical calculations and empirically predicted chemical shifts. MMCD also integrates LC-MS data collected under defined conditions and isotopomer masses for carbon to nitrogen [18]. This information can be combined to help identify the molecule in question. Recent developments in NMR software such as Bayesil [19], a web system that automatically identifies and quantifies metabolites developed at the University of Alberta (Canada), may also be useful. Similarly, the Metabolomics Fiehn lab offers an extensive list of NMR and MS related databases, chemometric tools, commercial and open-source software, as does the Bax Group. [20,21].

Undoubtedly, structure elucidation of any small organic molecule is a complicated process, and it is not surprising that spectroscopists may come to different conclusions from the same initial NMR data. This may be due to resonance overlap in NMR signals; incorrect chemical shift annotations or misjudged interpretation inferred from the presence or the absence of characteristic spectral features, no matter what the pulse sequence(s) or analytical tools are used [13]. This means a multi-faceted analysis and careful interpretation of the data are often helpful.

### 1.3. Gas Chromatography-Mass Spectrometry (GC-MS) Based Metabolomics

Mass spectrometry is the standard technique for the analytical investigation of molecules and complex mixtures. It is important in determining the elemental composition of a molecule and in gaining partial structural insights using mass spectral fragmentations. GC-MS based metabolomics can comprehensively resolve more than 300 compounds, but is limited to the analysis of compounds typically less than <500 Da. Additionally, the analysis of higher boiling point (generally polar) compounds must be made volatile via chemical derivatisation (which selectively alters known functional groups) to make them amenable to GC-MS analysis. One of the greatest advantages of GC-MS is that the electron ionization (EI) mode used in this technique is highly standardized and reproducible across GC-MS systems from different vendors. This allows the use of mass spectral libraries, such as the Agilent G1676AA Fiehn GC/MS Metabolomics RTL Library [22,23], or the publicly available Golm Metabolome Database (GMD) [24,25], National Institute of Standards and Technology (NIST) Mass Spectral Database [26] and the Wiley Registry™ (Hoboken, NJ, USA) of Mass spectral Data (now part of the Wiley Spectra Laboratory), 11th Edition [27]. Such libraries are however, limited in helping to solve the structure of unknown metabolites. To increase the number of identified metabolites in a metabolomics investigation, authentic standards of presently unregistered compounds are required [28]. In addition to the highly reproducible mass spectra, some commercially available libraries provide retention times or retention time indices (Kovats Indices) under standardized conditions for each metabolite [24], thus increasing confidence in the putative, metabolite identification. Alternative ionization methods such as chemical ionization (CI), which are “softer” approaches using less impact energy and resulting in less fragmentation and greater occurrence of the parent ion, are yet to be widely applied in metabolomics.

### 1.4. Liquid Chromatography-Mass Spectrometry Based Metabolomics

Liquid chromatography-mass spectrometry (LC-MS) is a complimentary analytical platform used to identify a diverse range of primary and secondary metabolites [29,30,31]. Liquid chromatography-high resolution mass spectrometry (LC-HRMS) metabolite profiling workflows using *state-of-the-art* high resolution instruments (see below) will generate many potential molecular formulas. In natural product research, such data can be cross searched with structural databases and taxonomic information, for example, via the Dictionary of Natural Products (DNP) [32] that holds more than 250,000 secondary metabolites. Several metabolite databases currently exist for the comparison of MS spectra, which include the Human Metabolome Database (HMDB) [33], METLIN [34] and MassBank [35]. These databases facilitate metabolite annotation through tandem MS/MS analysis. One of the major drawbacks with an LC-MS untargeted profiling approach is the number of “features” generated (this is sample dependent, but usually detects features anywhere from 500 to 5000). Features are made up of different salt and chemical adducts, clusters and in-source fragmentation products. The difficulty arises in assigning a chemical structure to all features; thus outlining the importance of structural confirmation (especially with respect to isomers). There are methods to deal with this issue. In a recent example by Reading et al., ion mobility-mass spectrometry (IM-MS) in combination with molecular modeling was used to assist in the structural isomer identification by measurement of gas phase collision cross sections (CCSs) [36]. The successful application of this approach to identify metabolites would facilitate resource reduction, and may benefit all research areas where metabolite annotation is required. In the same direction, initiatives towards precision or reproducible retention times in LC based on structure, for example in the field of natural product chemistry [37] have been instigated. An example of such an approach is “retention projection” which aims to facilitate the standardization and harmonization of retention times for identical compounds across laboratories [38].

### 1.5. Towards Standardization in Metabolomics

Metabolomics should be an unbiased, data-driven approach that interrogates biological systems with the aim of generating new hypotheses, thereby providing new biological knowledge. The initial effort for metabolomics standardization began over 10 years ago with the Metabolomics Standards Initiative (MSI) (based on a similar effort in proteomics), the standard metabolic reporting structure initiative (SMRS) and the Architecture for Metabolomics consortium (Armet), which focused particularly on NMR based metabolomics [39]. More recently, the European Union (EU) Framework Programme 7 EU Initiative “Coordination of Standards in Metabolomics” (COSMOS) developed a robust data infrastructure and exchange standards for metabolomics data and metadata [40].

Generally, the metabolomics community agrees that one of the grand challenges of metabolomics is the accurate identification of large numbers of metabolites, efficiently, via the use of various profiling techniques. While spectral standardization within a database is helpful, the diversity in acquisition is also beneficial for metabolite annotation (isomers), as it can highlight similar/dissimilar fragmentation processes across analytical conditions. Sumner et al. [39] reported the identification of known or unknown metabolites being generally governed by the following four guidelines:

1. Confident identifications are based upon a minimum of two different pieces of confirmatory data relative to an authentic standard.
2. Putatively annotated compounds (e.g., without chemical reference standards, based upon physicochemical properties and/or spectral similarity with public/commercial spectral libraries).
3. Putatively characterized compound classes (e.g., based upon characteristic physicochemical properties of a chemical class of compounds, or by spectral similarity to known compounds of a chemical class).
4. Unknown compounds—although unidentified or unclassified these metabolites can still be differentiated and quantified based on spectral data.

Making metabolomics data publicly accessible allows it to be used to justify researchers’ findings, increases the possibility of wider collaborations within the metabolomics community, and ultimately provides higher visibility and increased citation [41]. It is therefore important to make sure such publicly available data is as accurate as possible. How best to achieve this aim is however, not universally agreed upon and the subject of much discussion and debate.

This position paper is stemmed from one of several round-table discussions on the topic of “*Structure Elucidation*” from the inaugural Australian and New Zealand Metabolomics Conference (ANZMET) held at Latrobe Institute for Molecular Science, Latrobe University, Melbourne, Victoria, Australia on 30 March–1 April 2016.

## 2. Structure Elucidation by Nuclear Magnetic Resonance Spectroscopy (NMR)

NMR is a powerful analytical technique that is utilized widely in chemistry, biochemistry and structural biology. It provides a relatively simple method that allows for the simultaneous measurement of the ensemble of metabolites present in a solution with minimal sample preparation. The technique exploits the fact that every nucleus has a quantum mechanical property termed “*spin*” which can be conceptualized as a form of intrinsic angular momentum associated with a magnetic dipole moment. It should be noted that “*spin*” is rather an abstract term and it is misleading to imagine the nucleus as a small spinning object. The spin of a nucleus never changes, and is quantised, only taking values in increments of ½. The fact that nuclei, the electron and other charged particles are observed to be deflected by magnetic fields in a manner suggests they themselves have the properties of being small magnets and this can be usefully exploited.

When a sample containing NMR active nuclei is placed in a strong magnetic field aligned along the *z*-axis, the bulk sample magnetization aligns with the external field precesses about the applied magnetic field vector with a frequency characteristic of the nuclei in question, termed the Larmor frequency, and their exact electronic environment. By applying an electromagnetic pulse, in the radio frequency range, to, and in phase with the sample, the bulk magnetization vector can be made to rotate into the *x*/*y* plane where it continues to precess. By measuring the oscillation of the bulk signal in the plane and its decay back to alignment with the *z*-axis, characteristic frequencies of oscillation, termed chemical shifts (δ), commonly reported in units of parts per million (ppm), so as to be independent of the applied magnetic field, of a compound or compounds can be determined. As the chemical shift depends on the exact electronic environment surrounding the nucleus, a chemical shift can be diagnostic for a specific metabolite. The amplitude of the signal is—under suitable conditions, also proportional to the number of identical nuclei, and therefore the concentration of the metabolite, present in the sample.

The above description is a large oversimplification of NMR but serves as a model for understanding the method. For a complete description of NMR including descriptions of the method based on classical [42] and quantum mechanics [43,44] can be found elsewhere and so will not be discussed further here.

In contrast with MS, NMR is a rather insensitive but a universal detection method which also can provide absolute quantification, as such it is frequently used in metabolomics where it provides both quantitative and qualitative information [45]. It should be noted, that many applications of NMR—including those of interest to metabolomics researchers—can be understood and used to produce meaningful data, using little to no mathematics. In fact, modern NMR spectrometers allow their users to easily acquire and analyze complex spectra without the need for a complete or deep physical understanding of how the data are generated. What is generally required in metabolomics is the ability to use the NMR data to make useful and logical deductions and biologically relevant inferences as to the underlying biochemistry.

### 2.1. NMR in Metabolomics

NMR (and to a much lesser extent Magnetic Resonance Imaging or (MRI) has been the mainstay of metabolomics since the term “metabolome” was first coined in the late 1990s [46]. It could be argued that Nicholson and co-workers were actually carrying out NMR based metabolomics studies in the 1980s [47]. In some respects, the basic principles of NMR metabolomics have not changed considerably since that time.

NMR based metabolomics primarily makes use of those nuclei with a *non*-integer spin, such as ^1^H and ^13^C. Although each proton (or carbon atom) in a molecule typically has a different resonance frequency (chemical shift), the entire NMR spectrum is treated as a form of a biochemical fingerprint of a particular sample. High sample throughput allows for greater numbers of spectra to be obtained relatively quickly (acquisition of a “good” NMR spectrum takes on the order of ~ a few minutes using a modern automated system). Advanced multivariate statistical techniques such a principle component analysis (PCA) and partial least squares discriminant analysis (PLS-DA) are then used to detect changes in that fingerprint of particular groups that occur in response to factors such as disease or environmental influences [48]. Changes in the overall “*fingerprint*” can then be traced to changes in the levels of particular metabolites (or classes of metabolites) that are themselves indicative of changes in the underlying biochemistry in response to the original stimulus or stimuli. The use of such a method requires the answer to two pertinent questions, *how much sample is required in order to generate useful spectra* and *how can the peaks observed in said spectra be assigned*?

#### 2.1.1. How Much Sample Is Required?

NMR spectroscopy can detect metabolites low in concentration of 1–10 µM [49,50,51] and usually a sample volume of 600 µL is used [52,53]. There have however, been significant improvements in recent years via the use of stronger applied magnetic fields; this is advantageous as NMR signal intensity depends on the strength of the applied field. The use of cryoprobes also greatly improves signal to noise (*S/N*) of the spectrum by cooling the receiver coils to cryogenic temperatures thereby reducing the contribution of thermal and electronic noise to the spectrum [54]. The equipment costs involved in such approaches are high but some research groups have reported routinely obtained usable spectra using 100 µL at concentrations of only 50 µM, equating to 50 µg of sample [55] and structure elucidation from samples as low as 10 µg have also been reported [37]. Developments such as Dynamic Nuclear Polarisation (DNP), which boosts signal strength by an order of magnitude, are likely to push this limit even lower in future [56].

#### 2.1.2. Assigning Peaks

**Figure 1:**
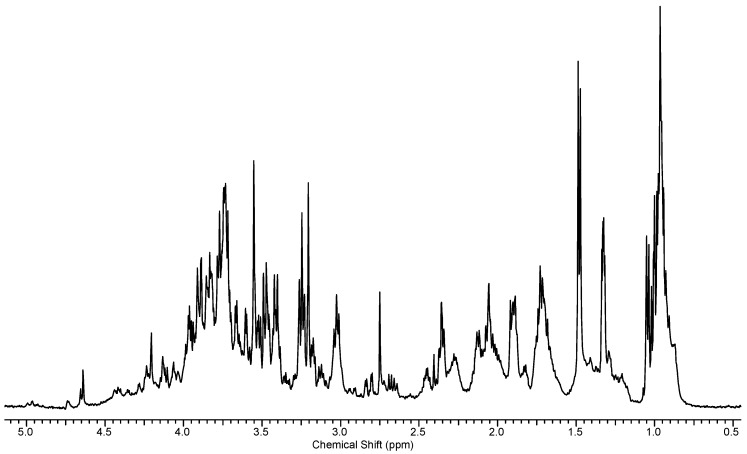
Nuclear magnetic resonance spectroscopy (NMR) of aqueous metabolites extracted from a sample of *Daphnia magna*. Complex overlapping peaks can be observed even in a shift range of just 0.5–5 ppm.

Since NMR/MRI based approaches allow for the quantification of metabolite concentrations of intact tissue, either in vivo [57] or ex vivo [58], the majority of metabolomics studies use one-dimensional (1D) proton (^1^H) NMR spectroscopy of cell or tissue extracts as the primary method of analysis. Because the ^1^H is abundant in biological molecules, and is also the second most NMR sensitive nucleus (after tritium), this method will be discussed here. A typical 1D ^1^H spectrum is illustrated below in Figure 1, in this case from an extract of the water flea, *Daphnia magna*. While many spectra are better resolved that that in Figure 1 they still show a multitude of overlapping peaks, which must all be assigned to specific metabolites present in the sample if useful data is to be obtained and interrogated.

The electronic environment of a nucleus is determined by its spatial arrangement to adjacent nuclei and the local environment of a nucleus shielding it, by a small amount from the external magnetic field. This means nuclei experience only a fraction of the strength of the external magnetic field and so have a correspondingly lower Larmor frequency. The result is a distinct chemical shift for each nucleus present in the molecule that exists in a unique local environment. Multiple protons display multiple peaks and each compound can potentially have multiple protons; this can equate to a large number of peaks in the resulting spectrum.

Shift values in ppm are used so as to be independent of the strength of the NMR spectrometer used to acquire spectra from the sample. Therefore, although sensitive to factors such as changes in pH and solvents (for the most part), the same compound in the same conditions will provide the same reproducible spectra. Although the use of higher field strength spectrometers (>900 MHz) can result in slight changes in chemical shifts and resolution in second order splitting, these are generally not an issue in metabolomics studies, which are usually carried out on instruments of between 300 and 800 MHz. Peak patterns from such instruments are thus usually diagnostic of the compound in question.

Since the early days of metabolomics a useful place to begin peak assignment is with tables of known chemical shift values from known standards. For example, a standard lactate NMR spectrum shows a doublet at around 1.2–1.3 ppm and a quartet at about 4.12 ppm. If these peaks are observed in the correct ratio then we can make a reasonable assumption that lactate is present. An additional peak at 4.5 ppm is the result of an OH group exchanging with residual water and the quartet is sometimes obscured by other overlapping resonances. The doublet is however, quite diagnostic. One can then analyze the rest of the spectrum and assign peaks until the entire spectrum (or the majority of it) is accounted for. While this method is crude and requires verification with known standards, it can be effective for working out what is present, if not for the structural assignment of unknowns.

**Figure 2:**
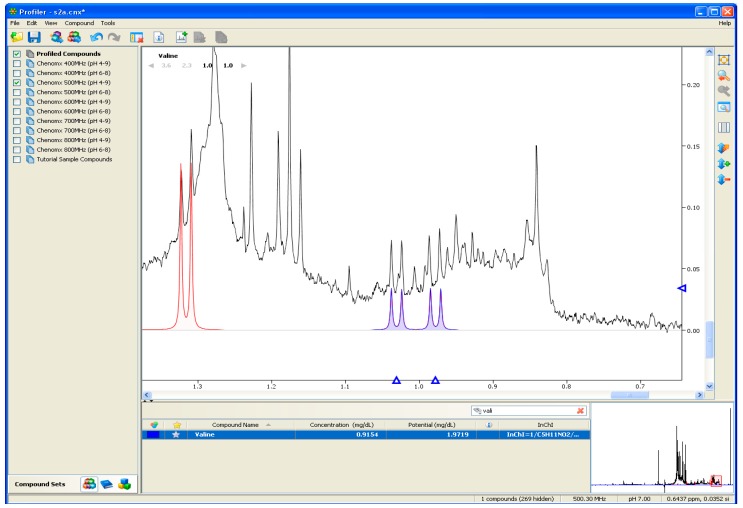
Section of a ^1^H NMR of soil extract (black) overlaid with NMR spectra of lactate (red) and valine (blue).

The development of large spectral libraries such as that of the HMDB [59], which contains the peak patterns and chemical shift values for thousands of metabolites, has greatly facilitated the identification and characterization of metabolites. For example, the development of software tools such as Chenomx NMR profiler™ (Edmonton, AB, Canada) allows the ^1^H spectra of the sample to be overlaid with spectra of known metabolites, so that over time a *pseudo*-spectra is built up that matches that of the sample [60], as shown in Figure 2. Although comparatively expensive and somewhat resource intensive, the Chenomx software can identify and quantify hundreds of small molecules. In its most recent update, Chenomx now offers data for compounds from the HMDB library. A similar tool, Mnova™ by Mestrelab Research (Santiago de Compostela, Spain) allows a wide range of functions on its desktop form and also comes in a tablet version.

Computational NMR approaches have advanced even further with the recent advent of automated processing techniques, such as Dolphin [61] for automatic targeted metabolite profiling using 1 and 2D ^1^H data and, Bayesil, a web based system that automatically identifies and quantifies metabolites using 1D ^1^H NMR spectra (at present only from plasma, serum or cerebrospinal fluid) [62]. Both programs have been shown to be able to take data from the user and assign peaks in the spectra better than the most experienced NMR expert [62]. Since sample identification is currently a major bottleneck in high throughput metabolomics it is likely that these forms of computational approaches will only gain greater attraction and appreciation in future, once the potential issue of the spectroscopist perhaps not trusting the computers judgment over that of a human operator is overcome.

### 2.2. Identification of Unknown Metabolites

Computational approaches work well when there is a known spectrum, or library of spectra to compare data to. In some cases, however metabolomics may lead to the discovery of an unknown metabolite, or metabolites, for which no library spectra exist—as previously discussed with respect to isolating a “pure” natural product. In one example, when the metabolic profiles of sexually active and inactive cells of the diatom *Seminavis robusta* were compared, a highly up-regulated metabolite was identified in the active type and later shown to be di-l-prolyl diketopiperazine, the first diatom pheromone [63]. Metabolomics was also applied to assist in the isolation and structure elucidation of 37 compounds including two anthocyanins from *Arabidopsis thaliana* [64].

In some cases a possible impression of what the structure of a new compound is likely to be may exist but in other cases there may be limited knowledge as to its identity. *How then can an unknown structure be identified*? While de novo structural determination via NMR has briefly been discussed, a full description will not be attempted here, since the topic can, and does, fill multiple books and papers [10,65,66,67,68]. The short answer is that it is possible to identify a metabolite by working out a chemical structure that is consistent with the observed spectrum and then confirming that structure with further analysis with other techniques such as MS and FTIR spectroscopy.

The first stage of identifying an unknown is to extract and purify a sample of it. Metabolomics often borrows techniques such as column chromatography, preparative HPLC, LC-NMR or LC-SPE-NMR [66,68] from the organic/natural product chemistry community to purify the sample in a reasonable amount to obtain a useful spectrum. Once a pure sample is obtained and a NMR spectrum acquired, one of the first steps in the structural characterization is to find the chemical shift of every ^1^H atom in the target molecule, a process usually referred to as resonance assignment. Since chemical shifts are sensitive to subtle changes (a ^1^H in an aromatic ring differs from a ^1^H attached to an electrophile such as O, N, etc.), these values can indicate which functional groups are present in the molecule and are thus are diagnostically useful.

Chemically *non*-equivalent nuclei in a molecule can also interact or couple with each other through bonds. The magnitude of this interaction (Hz) is smaller than a chemical shift (ppm) and is described by what is termed the scalar coupling constant (*J*). This interaction causes what is known as the *n* + 1 rule in the coupled system. The result is a multiplet (*n* + 1) peak pattern where the number of peaks for each coupled spin is one more than the number of nuclei coupled to it. For example, the doublet in lactate is caused by the fact that the methyl group protons on the terminal carbon are split by a single proton on the adjacent carbon, which itself is split into the quartet by the methyl group protons. The ratios of integrals comparing different signals arising from the same molecule provide an indication of the number of nuclei contributing to each peak. In the ^1^H NMR spectrum for lactate the areas under the doublet at 1.2–1.3 ppm is three times the size of the quartet, indicating that 3 protons contribute to the former and only 1 to the latter. Again, this information can be used to determine the structure of the molecule. A helpful illustration of ^1^H and ^13^C chemical shifts is available online [69,70].

The main disadvantage of 1D ^1^H NMR spectroscopy is signal overlap due to the narrow dispersion of ^1^H chemical shifts that occur between 0 and 15 ppm. In contrast, ^13^C NMR peak shifts occur in a chemical shift range of 250 ppm but since ^13^C is only about 1% of the total carbon in an ample the peaks are much smaller. Peak splitting usually does not occur in ^13^C NMR as protons are usually decoupled from the carbons via the pulse sequence, which means that ^13^C NMR spectra results in one peak for each carbon atom present in the molecule in a chemically distinct environment. This information can be added to that gained from the ^1^H NMR to help “narrow down” the list of possible structures and is certainly far more diagnostic. It is also possible to use a combination of ^1^H and ^13^C shift values (or those from other NMR visible heteroatoms such as ^14^N and ^31^P) to form multi-dimensional NMR spectra to reduce signal overlap and ease in metabolite identification.

### 2.3. Multidimensional NMR

Multidimensional or 2D NMR signals are a function of two frequencies rather than one, and the resulting data are plotted like a topographic map with cross peaks on the map indicating linked nuclei. Such experiments have somewhat “*off-putting*” names such as Correlation Spectroscopy (COSY), Heteronuclear Single Quantum Coherence (HSQC), Heteronuclear Multiple Bond Correlation (HMBC) and Nuclear Overhauser Effect spectroscopy (NOESY) but all provide important information on possible structure [8,43].

**Figure 3:**
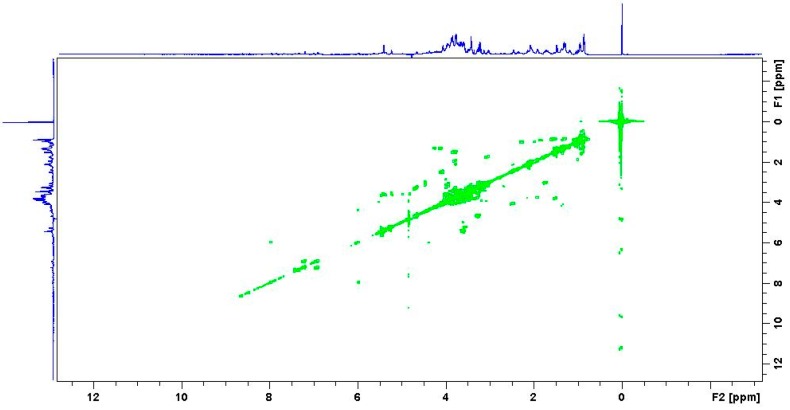
2D COSY ^1^H spectra of the *Daphia* sample from Figure 1 illustrating cross peaks.

A COSY spectrum indicates which ^1^H atoms are coupling with each other (both axes correspond to the ^1^H NMR spectra, and an example is illustrated below in Figure 3. The HMBC experiment provides correlations between protons and carbons that are separated by two, three, and, sometimes in conjugated systems, four bonds but direct one-bond correlations are suppressed. Conversely, in a HSQC experiment, the spectrum contains a peak for each unique proton attached to the heteronucleus being considered. The NOESY experiment correlates all protons close enough in 3D space and allows for the assignment of the relative stereochemistry of the molecules, assisting in the determination of probable isomers. Combining all this data allows the user to rule in and out certain structural configurations of the molecule under study.

### 2.4. Computational Structure Assignment

Calculating a chemical structure from NMR data is difficult even for an experienced NMR spectroscopist. Incorrect, published structures are not uncommon, and even the elucidation of small molecules often requires exhaustive spectroscopic analysis and/or full chemical synthesis to prove the correct structure. Recent developments in computer assisted structure determination from spectroscopic data have been shown to be useful to save time and improve accuracy. ACD/Structure Elucidator Suite is one of the more developed. Such systems can generate a complete set of all structures “fitting” the NMR correlations observed and other analytical information can then be used to narrow down structural possibilities [12,71]. Such approaches are likely to become more prevalent as metabolomics progresses. As the availability of public online metabolomics databases (such as HMDB and Chemspider) increase, spectroscopy, particularly (though not exclusively) NMR spectroscopy will likely allow metabolomic scientists greater insights into cellular metabolism [72]. The advent of public available metabolite databases containing full spectral data has reduced the work required to identify compounds and increasingly powerful computational approaches are likely to make such tasks increasingly easier and more routine. De novo absolute structure assignment will likely however, still, need human expertise for the foreseeable future.

## 3. Mass Spectrometry

Mass spectrometry (MS) measures the *mass-to-charge* ratio (*m*/*z*) of individual molecules present as charged ions. Modern high resolution mass spectrometry (HRMS) is an essential tool in metabolomics research, providing many analytical advantages, including the ability to measure a broad range of analytes at high mass resolving power with high mass accuracy across wide mass ranges and providing molecular specificity and high sensitivity in detection. Even with these excellent features, MS is limited in its ability to completely elucidate the 3D structure of a molecule and is usually unable to distinguish enantiomers.

Structural information, including molecular connectivity, can be gleaned by employing different fragmentation methods in tandem MS approaches (MS/MS). When HRMS is used, the molecular formula can generally (and even unambiguously), be identified using ultra-HRMS (uHRMS) and/or by using a combination of heuristic filters [73] to keep only chemically coherent molecular formulae from all possible hits within a given ppm range. As with NMR, for high-throughput metabolomics measurements there is a clear requirement to be able to identify many biomolecules from complex mixtures; MS methods that couple HRMS to different ionization sources, multi-stage MS/MS and extensive databases of standards enhance the ability to identify any individual known metabolite in a sample are the best methods to use in metabolomics [74]. This approach provides increased depth of coverage and also generates important information that aids in structural elucidation. 

To provide guidance to researchers, a number of initiatives have generated a series of identification standards and proposed guidelines for reporting structural identification by MS. The Metabolomics Society MSI task group were introduced in 2007 [39]. More recently the Swiss Federal Institute of Aquatic Science and Technology (Eawag) has introduced a series of levels to provide confidence of assignment when identifying metabolite by HRMS [75].

MS alone is not able to unambiguously identify a molecule and must rely on complementary sources of information (e.g., chromatographic retention time, MS/MS, additional UV/IR spectroscopy, CD or NMR etc.). The currently accepted analytical standard is an isolated and authentic chemical or metabolite to which analytical data can be directly compared. With the above in mind and the large number of mass spectrometers and approaches available to researchers’ understanding the fundamental principles of mass spectrometry are critical to obtain meaningful data.

### 3.1. Types of Ion Sources

A mass spectrometer is composed of the ionization source, mass analyzer and detector, each having quite distinct and vital roles in the cumulative production of interpretable structural information and ultimate identification of discrete species in complicated biological matrices. There are currently a plethora of ion sources used in modern mass spectrometry, with a large number of ambient ion sources developed recently. Of principle consideration in ionization methods remain, (i) the physio-chemical attributes of the analyte(s) in question (e.g., ionizability, thermal lability) and (ii) the magnitude of internal energy transferred through the ionization process. The critical selection, and understanding of generated information from, ion sources is imperative. Classification of ion sources are organized by the nature of the substance and such methods are wide and varied. Some of the more common are EI, chemical ionization (CI), field ionization (FI), atmospheric pressure chemical ionization (APCI) and atmospheric pressure photoionization (APPI); and desorption, featuring field desorption (FD), electrospray ionization (ESI), matrix-assisted laser desorption/ionization (MALDI), plasma desorption (PD), thermospray ionization (TS) but there are many others. It is important to note that, while being sensitive, MS ionization (and thus detection) is strongly compound-dependent and thus the choice of an adapted ionization method and polarity mode for a given metabolite is perquisite for its effective analysis.

An additional quality of ionization method is its inherent sub-classification of *hard* or *soft*. Hard ionization (such as that produced by the high 70 eV ionization energy of an EI source) is accompanied by extensive fragmentation often, but not always resulting in the absence of a molecular ion. Whereas, soft ionization (such as that produced by CI and ESI) produces little fragmentation and usually conserves the molecular ion (parent ion). Current preferred sources for the analyses of biomolecules include conventional high-vacuum EI, CI and traditional MALDI, and those centered on atmospheric ionization in both positive and negative ion modes such as ESI, nanoESI, APCI and APPI and atmospheric pressure MALDI [76].

Electron ionization (EI) is a highly reproducible and data-rich technique predominantly in GC-MS systems, favoring both insoluble and non-ionic analytes of low- to medium-polarity. While the inherent reproducibility of EI enables unambiguous identification of analytes, the fragmentation patterns EI generates can be influenced by complex rearrangements limiting accurate peak assignments. The use of the soft ionization source, chemical ionization (CI) in place of EI can provide critical information pertaining the molecular ion, typically either absent or of too low abundance when an EI source is employed. Though traditionally used in GC-MS systems, recent advances such as supersonic molecular beam (SMB) LC-MS and direct-EI LC-MS demonstrate a viable alternative to EI sources while retaining the benefits [77]. Modified GC-MS systems incorporating SMB technology, also termed “cold EI,” generate a higher abundance of molecular ions (though current use seems limited to the analysis of hydrocarbons and petrochemical products) and its applicability to the analysis of biomolecules remains of significant interest.

Electrospray ionization (ESI) is now the method of choice for the analysis of biomolecules such as: proteins, polymers, biopolymers; polar/basic/charged small molecules, particularly due to its ability to study *non*-volatile and thermally labile analytes of *f*mole concentrations in μL-size samples [78]; to ionize proteins without denaturization; low flow rates (nL–μL/min) and compatibilities with a range of polar solvents. Nanoelectrospray ionization (nanoESI) offers several additional advantages including enhanced mass sensitivity, superior ionization efficiency and reduced sample consumption (analyte concentration is more critical than injected sample volume). ESI is now widely used for the analysis of many small molecules where it produces singly charged ions for a vast majority of polar to medium polar metabolites in natural product research or in bodily fluid analysis [74].

Matrix effects and ion suppression remain significant obstacles inherent to ESI ion sources. The analysis of compounds in complex matrices (*clear-cut* in causing ion suppression, due to presence of other endogenous matrix species likely to cause large ionization interferences) may require use of APCI/APPI sources in concert or in place of ESI, due to their characteristic tolerance of matrix effects such as high buffer concentrations. In addition, APPI sources are useful in analysis of both non-polar compounds and others not ionizable by either APCI or ESI sources [79]. The trend towards the use of multi-mode ion sources (parallel ionization techniques) has assisted in both shortening analysis time and acquiring structural information from a larger range of analyte classes [80]. It is also interesting to note a recent study demonstrating neither ESI source design, nor source geometry, imparts any observed benefit in reducing matrix effects [81].

Current knowledge pertaining to ion formation mechanisms within ESI sources is rather vague. A more comprehensive understanding of the mechanisms (such as the mechanistic nature of gas-phase ion formation) guided by models as the ion evaporation model (IEM), charged residue model (CRM) and chain ejection model (CEM) is needed in order to enhance both our interpretation of ion formation, and by extension, our ability to enable more purposeful and specific customization of MS parameters to target analytes (or compound classes) of interest [82].

Matrix-assisted laser desorption/ionization is a high-sensitivity *non*-destructive vaporization and ionization technique employed for the analysis of proteins, peptides and low-mass molecules. MALDI requires that an analyte first be co-crystallized with a large molar excess of matrix compound (e.g., UV-absorbing weak organic acid) followed by vaporization of the matrix through laser excitation and ablation [83]. While MALDI has seen increased popularity in biomolecule analysis and imaging areas, the technique’s potential in metabolomics is yet to be fully realized. It is hampered by significant signal suppression from interfering low-mass ions (<500–700 Da) when conventional matrices (e.g., 2,5-dihydroxybenzoic acid (DHB) and α-cyano-4-hydroxycinnamic acid (CHCA) are used). In combating this limitation, the use of porous silicon chips/matrices with reduced background signals have been employed, with notable examples being matrix selection based on Brønsted-Lowry acid-base theory [84] and the novel “proton sponge” matrix [84].

Direct molecular profiling by MALDI using nanoparticles (including those composed from diamond, graphite, silver and or gold) has been successfully applied in metabolomics studies [85] and has aided in significantly reducing chemical interactions with biological tissues in the ground state [86]. Metabolic imaging of biological tissue without the need for matrix deposition (i.e., LDI) has also been successfully demonstrated [87]. In addition, laser wavelength has been highlighted as a critical parameter in both spatial resolution and overall sensitivity of MALDI and LDI methods [87,88].

Ambient mass spectrometry using direct analysis in real-time (DART) ion sources presents a powerful and exciting innovation in high-throughput metabolome analysis, allowing for direct ionization of samples with minimal pre-treatment. Several notable studies have shown the broad applicability and accuracy of DART ion sources in metabolic profiling [89,90,91]. Indeed, DART may soon become a mainstay in the ion sources of choice for metabolomics-centered studies.

### 3.2. Mass Analysis

Once ionized, ions are measured by the mass analyser (see Table 1) with the ability to distinguish one mass peak from an ion close in mass described by both mass resolution and resolving power (RP). Mass resolution is defined as the degree of separation between two adjacent ions observed in the mass spectrum (Δ*m*) at Full Width Half Mass (FWHM) of the peak and RP is the inverse of mass resolution defined as the nominal mass (*m*) divided by the difference in masses (Δ*m*). Higher mass resolution allows easier identification of contributing ions and exclusion of interference from the presence of other chemical entities. Higher mass RP is essential for high mass accuracy whereby a higher RP allows identification of the centre of a peak and determination of mass error. Mass error is the difference between the observed mass and theoretical mass of a given ion; lower mass error allows higher confidence assignment of molecular formula aiding identification. Within the high throughput metabolomics context where analysis of large numbers of complex samples is desired a high mass resolution detector with low mass error is essential to provide confident assignment of molecular formula. The most common mass analyzers of modern HRMS are Time-of-Flight (TOF) capable of <5 parts per million (ppm) mass error and FT instruments, both Orbitrap and Ion Cyclotron Resonance (ICR) both routinely capable of mass errors <2 ppm.

**Figure 4:**
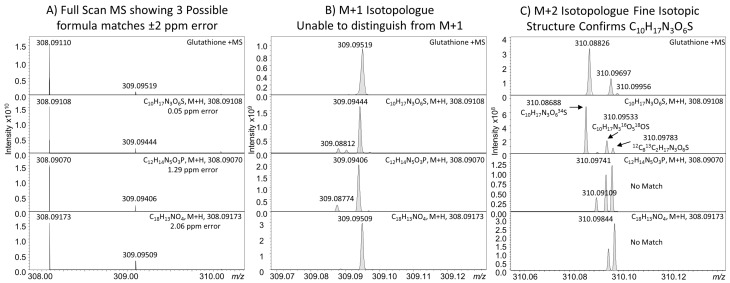
Identification of molecular formula using uHRMS in combination with isotopic fine structure: (**A**) Full Scan MS^+^ spectra for a glutathione standard showing three calculated formula matches within 2 ppm mass error; (**B**) Expansion of *m/z* 309.07–309.13 showing M + 1 isotopologues and inability to determine parent formula; (**C**) Expansion of *m/z* 310.06–310.13 showing M + 2 isotopologues with calculated isotopic fine structure match of ^34^S, ^18^O and ^13^C_2_ isotopologues to C_10_H_17_N_3_O_6_S matching the glutathione standard, other formula fine structures do not match the same isotopologue profile.

The presence of either isomers (structural or stereo) or near-isobaric chemicals with nominally the same or similar masses confounds the interpretation of the resulting mass spectra. Near isobaric compounds can be distinguished by the use of uHRMS employing higher mass RP, which at the extremes can be used to provide unambiguous identification of molecular formula by measurement of the isotopic fine structure of molecules containing elements with multiple isotopes [92]. To conduct these measurements, RP > 300,000 are required and are only possible using FT instruments, including FT-ICR and the latest Orbitrap MS instruments, a contrived example for identification of a glutathione standard is shown in Figure 4, [93]. The current limitations are that these types of analyses are slow, requiring longer scan times to achieve the desired resolution and are only feasible on relatively abundant ions. Both decreases in scan times and increases in instrument sensitivity will see the application of uHRMS measurements increase in metabolomics.

### 3.3. Tandem Mass Spectrometry

**Figure 5:**
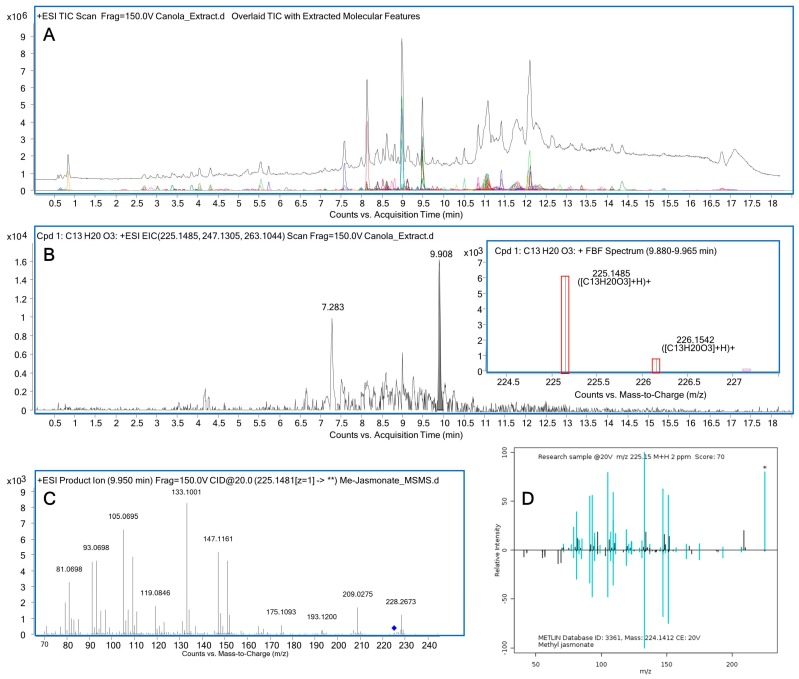
Dereplication and identification strategy using Liquid Chromatography Mass Spectrometry: (**A**) Reverse Phase ESI^+^ Total Ion Chromatogram with overlaid Extracted Molecular Features of Canola Extract; (**B**) Extracted Ion Chromatogram of the molecular formula corresponding to the plant hormone methyl jasmonate with inlaid Find-by-Formula Spectrum showing observed and predicted isotopic abundance for the feature at 9.880–9.965 min; (**C**) Product Ion spectra of methyl jasmonate using collision voltage of 20.0 eV; (**D**) Spectral match of methyl jasmonate MS/MS spectra to the METLIN Database allowing Putative identification.

Even the ability to identify molecular formula to a high degree of accuracy is still insufficient information to provide the confident identification of a structure, therefore further MS/MS [94] is used to generate additional evidence to identify individual compounds by fragmentation analysis. An example of the identification of methyl jasmonate from canola extracts is shown in Figure 5, demonstrating the use of accurate mass match and confirmation by MS/MS spectral match to the METLIN database. For this application hybrid instruments combining one or more different mass analyzers are generally used; typically a mass selective quadrupole or linear ion trap coupled to a collision cell will be operated with a higher mass resolution analyzer such as a TOF or FT. Precursor ions are energetically fragmented using one or more of a number of techniques including Collision Induced Dissociation (CID) [95], Higher Energy Collisional Dissociation (HCD) [96], Electron Transfer Dissociation (ETD) [97], Electron Capture Dissociation (ECD) or Electron Induced Dissociation (EID) [98], Sustained Off-Resonance Collision-Induced Dissociation (SORI-CID) [99] and Ozone Induced Dissociation (OzID) [100].

The most common techniques for fragmentation analysis will likely remain CID, HCD and EI due to broad applicability and speed. Individual chemicals fragment in unique manners generating combinations and relative abundances of product ions that can be compared to an authentic standard to identify the precursor ion or in the case of molecules that contain common chemical moieties to in silico structural databases to provide a tentative annotation. Identification may be difficult due to low fragment ion abundance or interfering ions for individual metabolites. To increase sensitivity, selected reaction monitoring (SRM) and multiple reaction monitoring (MRM) type experiments can be employed using instruments capable of MS/MS and MS^n^ but the trade-off is an inability to monitor wide mass ranges. These are typically actualized in low mass resolution instruments that employ triple quadrupole (*QQQ*) or ion-trap type mass analyzers. These provide targeted and fast data when coupled with authentic standards and orthogonal separation prior to mass analysis, thus have limited application in discovery and high throughput untargeted metabolomics. For untargeted profiling, acquisition of MS/MS spectra on virtually all metabolites in deep metabolome annotation studies can be achieved using two differentiated acquisition modes: Data-Dependent Analysis (DDA) and Data-Independent Analysis (DIA). DDA provides high-quality data but fewer MS/MS acquisitions during the run, whereas DIA allows better metabolite coverage at the expense of losing the direct link with the parent ion [101].

### 3.4. Chromatography-Mass Spectrometry

When MS is coupled to chromatographic separation prior to ionization and detection, large increases in sensitivity and depth of coverage are gained by suppression of the biological matrix effect. The two most common forms of chromatographic approaches are Gas-Chromatography and Liquid Chromatography. Orthogonal separation allows retention time (RT) to be coupled to mass providing a higher degree of confidence in identification. In Figure 5 more than 2000 molecular features extracted from canola extracts are demonstrated. Searching the extracted features for specific molecular formula allowed tentative identification of the plant hormone, methyl jasmonate. Further, matching of RT, mass and fragmentation data to authentic standards provides enough information to propose putative and even confirmed structural assignment, allowing downstream interpretation of the acquired data. Matching of MS/MS spectra acquired from tentatively identified methyl jasmonate to the METLIN database allowed putative identification of the hormone.

### 3.5. Ion Mobility-Mass Spectrometry

Separation and detection of ions in the gas phase is also possible via ion mobility and a number of different ion mobility MS (IM-MS) approaches have been described [102,103]. First introduced in the 1960s it was not until 2006 that the first commercial IMS-MS instrument was introduced by Waters, utilizing travelling-wave IMS (TWIMS). Since then a number of vendors have introduced different ion mobility systems and recent advances have seen separation resolutions of up to 400 in Trapped Ion Mobility systems (TIMS) [104,105]. In such systems ions are separated based upon differing mobilities in an electric field and atmosphere. Depending upon the IMS-MS approach used an ion’s gas phase structure can be studied by measurement of collisional cross section (CCS). If an ion’s structure and CCS is significantly different it can be separated from another ion of similar mass raising the ability to separate structural isomers and in certain applications even enantiomers. For metabolomics applications IMS-MS coupled to LC or GC promises a number of significant advantages including improved *S/N* ratio, higher peak capacity and the ability to separate isomers. At present the approach is yet to reach mainstream metabolomics and no large-scale libraries of CCS are available to aid in identification but the technology may soon become an essential mainstay of metabolomics research due to enhanced abilities to separate and identify molecules.

### 3.6. Metabolite Annotation

There are a number of online databases that include high resolution MS spectra and also in some cases MS/MS fragmentation spectra of endogenous and exogenous molecules that are useful for identifying metabolites and chemicals, including: LIPIDMAPS [106], MassBank [35], mzCloud [107], and others discussed above. Further compound databases that can be searched for accurate mass formula matches and in silico predictions and molecular fragmentation include PubChem, ChEBI, in vivo/in silico Metabolites Database (IIMDB) [108], and the LipidBLAST library [109].

In recent years, significant effort has been placed on developing novel in silico fragmentation and prediction algorithms resulting in a number of different approaches and software, Competitive Fragment Modelling (CFM) [110], MAGMa [111], CSI:FingerID [112] Highchem Mass Frontier™ (Bratislava, Slovakia),and MetFrag [113]. Regular competitions through the Critical Assessment of Small Molecule Identification (CASMI) [114] drive the development and testing of new methodologies and approaches. Recently, for example using CFM and spectral matching (Tremolo), an in silico fragmented database of more than 220,000 natural products [115] was used to enhance the precision of annotations in a dereplication workflow where only molecular formula and taxonomy cross searched assignments are used.

### 3.7. Organisation of MS/MS Data in Molecular Network to Enhance Annotation

Other approaches that can considerably assist metabolite annotation can be obtained through MS data organization and the major massive MS/MS data organization tool for this is the Global Natural Products Social molecular networking platform (GNPS) [116]. GNPS allows researchers to organize MS/MS data as spectral or molecular networks (MNs). In such networks, closely related MS/MS spectra and chemical related molecules represented as nodes corresponding to their detected *m*/*z* feature are linked closely. This way of representing a metabolome is radically new and offers a real paradigm shift in metabolome annotation. The validation of single annotation even suing just an in silico match with a given metabolite is improved by the fact that many closely related compounds match similar structures. Application of such methods for the putative and tentative annotation of unknowns have been validated for example in natural product research by targeted MS isolation of new alkaloids after prediction based on MN analysis [117].

## 4. Fourier Transform Infrared Spectroscopy (FTIR)

Multiple Fourier Transform InfraRed spectroscopy (FTIR) techniques such as transmission, attenuated total reflection, microspectroscopy, have been used to metabolically characterize bacteria, yeast and other microorganisms [118]. Briefly, FTIR is a method of measuring the infrared absorption and emission spectra of pure compounds and/or mixtures of compounds. FTIR takes advantage of the fact that molecules absorb specific frequencies that are characteristic of their structure (i.e., bond type and presence of specific moiety). As a result, a unique fingerprint representing the spatial frequency as a measurement of a peak at a given wavenumber is produced (ranging from ca. 500 to 3700 cm^−1^). The wavenumbers are assigned based on the absorption radiation and the transition energy of a specific bond type or moiety vibration. This transition energy is determined by the shape of the molecule and its potential energy, the masses of the atoms, and the associated vibrational coupling which in turn determines the type of vibrations that are associated with a particular mode of motion and bond type (such motions can either be observed as stretching, scissoring, rocking, wagging, and twisting) [119]. Several research groups have focused on the use of FTIR for authentication, identification, and classification of compounds and/or metabolites. This is primarily because FTIR offers several advantages over MS and NMR based techniques for structure elucidation, such as being relatively quick, simple, and *non*-destructive [120]. As such, FTIR is a powerful spectroscopic technique that provides insight into the functional groups as well as the chemical composition of isolated metabolites or complex metabolic samples.

**Figure 6:**
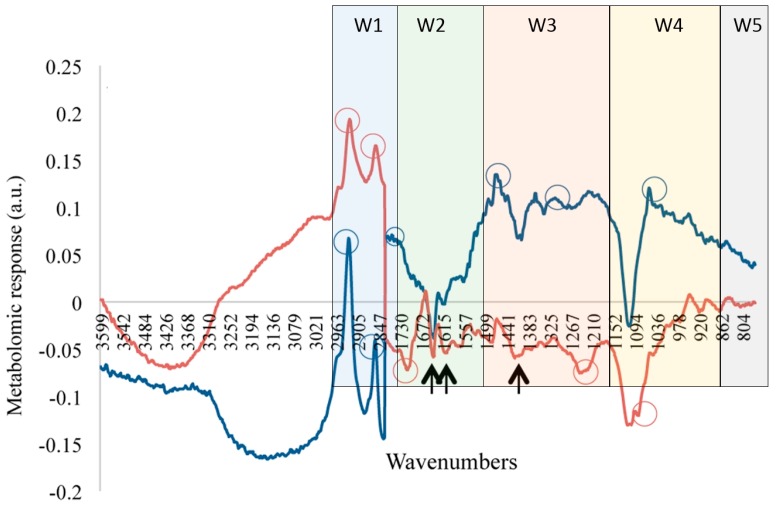
Comparison between the FTIR Response Spectra of *E. coli* and *L. innocua* at the surfactants concentrations of *N*-tetradecyltropinium bromide that resulted in 100% mortality. Note: a.u. stands for “arbitrary units”. Arrows correspond to the wavenumbers referred to peptidoglycan while circles to the wavenumbers referred to the major response peaks in each species. Blue, *L. innocua*; Red, *E. coli*. The synthetic stress of the metabolomic stress response are defined by the following FTIR regions: fatty acids (W1) from 3000 to 2800 cm^−1^, amides (W2) from 1800 to 1500 cm^−1^, mixed region (W3) from 1500 to 1200 cm^−1^, carbohydrates (W4) from 1200 to 900 cm^−1^ and typing region (W5) from 900 to 700 cm^−1^. Adapted from Corte et al. [125].

FTIR has been applied in microbiological studies for the analysis of the whole cell metabolome [121,122,123,124,125]. Corte et al. [125] developed a FTIR-based bioassay in order to determine the presence and the extent of cellular stress, with the rationale that stressing conditions can alter the cell metabolome. Similar approaches have been used to assess microbial environmental stress [126] and to identify the types of molecules involved in stressed mammalian [127], yeast cells [128] and to investigate preharvest sprouting in wheat and barley [129]. Figure 6 illustrates the effect of the surfactant *N*-tetradecyltropinium bromide at a concentration of 0.4 mM and 0.6 mM to *L. innocua* and *E. coli*, respectively [125]. This spectrum is obtained as the difference between the normalized spectrum of cells subject to the tested surfactant and that of the control maintained in water in the same experimental conditions. The spectra were then evaluated based on synthetic stress indexes of the metabolomic stress response, which is calculated as the Euclidean distances of the FTIR spectra under stress and the control FTIR spectra [130]. The stress indexes of the FTIR spectra were defined as the regions relating to fatty acids (3000 to 2800 cm^−1^), amides (1800 to 1500 cm^−1^), a mixed region (1500 to 1200 cm^−1^), carbohydrates (1200 to 900 cm^−1^) and typing region (900 to 700 cm^−1^). As the five spectral regions differ in length, the data was first scaled to the length of the whole spectrum in order to make the different regions comparable.

According to the FTIR analysis (Figure 6), the whole cell response to the surfactant *N*-tetradecyltropinium bromide that resulted in 100% mortality was concentrated in the fatty acid and amide regions. These responses were detected at 2920 cm^−1^ and 2852 cm^−1^, which represents the asymmetric and symmetric stretching (ν (CH_2_)) of the fatty acids chains. Furthermore, minor variations in the spectra were detected for *E. coli* in the fatty acid region when compared to the *L. innocua*. This was evident by the negative peak at 1708 cm^−1^ (ν (C=O) H-bonded). Other potential responses were detected for both species in the mixed region at ca. 1240 cm^−1^ (ν (P=O) asymmetric in phospholipids) for *E. coli*, while in *L. innocua* at ca. 1460 cm^−1^ (δ (CH_2_) of lipids and proteins) and at ca. 1310 cm^−1^ (Amide III). Lastly, the region representing carbohydrates of *E. coli* presented a negative peak at 1085 cm^−1^ (symmetric ν (P=O)) in nucleic acids and phospholipids, while *L. innocua* displayed a positive response around 1050 cm^−1^ (stretching of phosphate ester). This approach—while not explicit in the identification of metabolites—does provide insight into the stress response of chemicals to the whole cell.

Winson, et al. [131] employed diffuse-reflectance absorbance FTIR as a method of chemical imaging and rapid screening of biological samples for metabolite overproduction, using mixtures of ampicillin with *E. coli* and *Staphylococcus aureus* as model systems. This approach enabled the rapid identification and quantification of ampicillin in complex matrices. Similarly, Olson, et al. [132] used gas chromatography-based separation coupled with a FTIR detector in order to characterize the intermediate metabolites in the microbial desulfurization of dibenzothiophene.

A analogous study was conducted by Guitton, et al. [133], where GC-MS was used to putatively identify fentanyl metabolites in rat microsomes. Fentanyl is a synthetic opiate used for surgical analgesia and sedation that undergoes extensive hepatic biotransformation. GC-FTIR, while note routinely used for structure elucidation, was then used to validate the fentanyl metabolites identified based on known FTIR spectra. Lewis et al. [134] investigated the application of FTIR as a high throughput and cost effective method for identifying biochemical changes in sputum as biomarkers for detection of lung cancer. In doing so, a panel of 92 infrared wavenumbers were found to be significantly different between cancer and “*normal*” reference sputum spectra (where sputum is a mixture of saliva and mucus coughed up from the respiratory tract). These changes were associated with putative changes in protein, nucleic acid and glycogen levels in tumors. In conclusion, Lewis et al. observed five prominent significant wavenumbers at 964 cm^−1^, 1024 cm^−1^, 1411 cm^−1^, 1577 cm^−1^ and 1656 cm^−1^ that separated cancer from normal FTIR. This suggests FTIR might have high enough sensitivity and specificity in diagnosing lung cancer, along with further development and validation, has the potential to be used as a non-invasive, cost-effective and high-throughput method for screening.

Such approaches described herein rely on understanding the basic principles of compound elucidation using FTIR. This is best encapsulated in Table 2 below. Furthermore, there are numerous FTIR spectral datasets and reference libraries to which compound identification and comparisons can be made relating to species of interest (i.e., NIST, US EPA AEDC/EPA spectral databases). This includes a vast number of metabolites and pharmaceutical chemical compounds used in metabolomics studies. As noted by a vast number of authors, the principal advantage of FTIR is its rapidity and ease of use, compared to advanced analytical procedures such as MS and NMR. However, FTIR based approaches are considered less robust and pre-processing of plate cultures and the preparation of samples in general greatly influence the quality of spectra obtained [135].

## 5. Future Perspectives

The ability to identify molecules in metabolomic data is tied to three features: (1) the sensitivity of the instrument; (2) the size of existing spectral libraries; and (3) the skill of the scientist in making sense of, and interpreting, the data. In NMR, where the lower limit of sensitivity is typically 5–10 µM, the number of detectable compounds is usually between 40 and 200, depending on the biological material being analyzed. If the NMR spectra are relatively simple, such as those measured from serum, cell extracts, saliva, milk, juice or wine (with no more than 40–60 compounds), it is often possible to identify 90%–100% of the compounds using standard NMR spectral libraries [136,137]. When NMR spectra are crowded and complex, such as those from urine, environmental samples or cell growth media (with 100–200 compounds each), it is usually only possible to confidently identify 50%–60% of the chemicals [3]. In mass spectrometry, especially LC-MS, the lower limit of sensitivity is often 5–10 nM. Therefore, the number of detectable mass *features* is typically between 5000 and 20,000. In most cases, the number of confidently identified compounds (where both MS and MS/MS matching is done using comprehensive spectral libraries) is typically less than 300 and rarely more than 500 [138,139,140]. In other words, for LC-MS, just 4%–5% of the peaks can be identified in most biological samples. This needs to improve if metabolomics is to progress.

Over the coming years we can expect to see the development of instruments and techniques, in both NMR and MS, that will greatly improve their sensitivity and their lower limits of detection. In NMR, these will include such developments as increased field strengths (to 1.2 GHz and higher), selective isotopic labeling and improved probe designs that should allow sub-micromolar sensitivity to perhaps become fairly routine [141]. They will also include the development of dynamic nuclear polarization (DNP) NMR using conventional or Sabre-Sheath methods [141,142] that may ultimately lead to the detection of nanomolar levels of metabolites.

In LC-MS, continued improvements in separation techniques, e.g., with the generalization of ultra-high performance chromatography (UHPLC), detector technology and ionization methodologies suggest that single cell metabolomics with picomolar sensitivity should soon be widespread [143]. While such improvements are welcome, they will also lead to challenges with metabolite identification. *The data discussed above suggests that for every 10-fold improvement in sensitivity, there is typically a 2-fold increase in the number of “unknown” compounds that cannot be identified. What are these unknowns*? In some cases, the unknowns are actually known compounds but their spectra (NMR or MS) are not yet deposited into publicly available spectral libraries. These are often called the “known unknowns.” In other cases, these features are truly unknown compounds for which no structure or literature description exists. These compounds are sometimes called the “unknown unknowns” or the “dark matter” of the metabolome [144].

As to how to address the problem of identifying the “known unknowns” that answer is fairly clear. Additional NMR and MS/MS or EI-MS spectra need to be collected for more compounds and these spectra need to be deposited into public databases. Currently the largest NMR spectral databases designed for metabolomics (such as BMRB and HMDB) contain NMR spectra of fewer than 900 compounds [59,145]. The largest repositories of MS/MS data designed for metabolomics (such as METLIN and MoNA) contain MS/MS spectra of fewer than 20,000 compounds, many of which are di- and tripeptides [146,147]. The largest repositories of EI-MS data (such as the NIST library) contain spectra for more than 40,000 compounds, but many of these are not metabolites [148].

Whether it is for NMR, MS/MS or EI-MS, the key challenge with spectral collection is the lack of availability of pure reference compounds. If one excludes peptides (di, tri- and tetrapeptides), the number of commercially available, pure “natural products” is probably no more than 3000 compounds. If one includes synthetic compounds that are found in foods, drugs, cosmetics and industrial products, the total can be pushed to perhaps 20,000 compounds. Given that it costs >$600,000 and took 2 years to acquire 1000 compounds and measure their NMR, MS/MS and EI-MS spectra in the HMDB, acquiring a collection of 20,000 compounds and measuring their NMR, MS/MS and EI-MS spectra would cost millions of dollars and potentially take decades of work. Nevertheless, such an effort is well worthwhile. While it may not be possible for a single laboratory to do this, the idea of “crowd-sourcing” or “community exchange” is particularly appealing. The idea behind crowd-sourcing is to have thousands of individual laboratories or investigators randomly collect and deposit spectra of purified compounds into common, open-access repositories. This is the basis to databases such as GNPS, MetaboLights and MassBank of North America (MoNA) [116,147,149]. Certainly, if scientific journals and publishing houses were to buy into this crowd-sourcing concept then the cost of such an effort could be greatly reduced and the time to complete the process could be significantly shortened.

The collection of hundreds or even thousands of new NMR spectra will likely solve most of the “unknown” problems for the field of NMR-based metabolomics. However, the “unknown” problems will likely persist for MS-based metabolomics. Based on several lines of evidence [139,140,150], even if thousands of metabolically meaningful MS/MS or EI-MS spectra could be collected and deposited over the coming years, it is still unlikely that the fraction of identified metabolites from LC-MS/MS or EI-MS studies would rise much above 20%. This is because most of the unknowns in LC-MS/MS or EI-MS are either compounds that are far too difficult to isolate or synthesize or they are compounds that have not yet been described or discovered. That is, they are the “unknown unknowns.”

Solving the problem of the “unknown unknowns,” especially for LC-MS/MS and EI-MS-based metabolomics will require greater intellectual effort than a simple *brute-force* metabolite synthesis or spectral data collection. In particular, it will require the development of sophisticated computational methods that can predict or interpret MS/MS and EI-MS spectra using known (or predicted) chemical structures. There are more than 300,000 natural products that have had their structures described [115] and more than 60 million synthetic compounds that have been synthesized and have had their structures deposited in PubChem [151]. Rather than trying to isolate, resynthesize or re-acquire these compounds (which would cost billions of dollars), it would be far easier to predict their MS/MS or EI-MS spectra or to see if observed MS/MS or EI-MS spectra fit to existing structures. As already mentioned, over the past few years some significant developments in MS/MS spectral prediction and MS/MS spectral interpretation have taken place. These include the development of MetFrag [113] and the CFM-ID suite for predicting both EI-MS and MS/MS spectra [148,152] as well as the development of LipidBlast for predicting MS/MS spectra of lipids [153]. They also include the creation of tools such as MAGMa [111], CSI:FingerID [154], and IOKR [112] which match observed MS/MS spectra to chemical structures via fingerprint analysis. These predictive tools are now being exploited to perform novel metabolite identification. In one particularly impressive example, MS/MS spectra were predicted via CFM-ID for all of the compounds in the Dictionary of Natural Products [115]. By constructing a spectral similarity network and using sophisticated spectral matching comparisons it was possible to use this network of predicted MS/MS spectra to identify a number of previously unidentified compounds [155].

Of course, not every metabolite that is detectable by LC-MS today (or in the future) is likely to be something already in a chemical structure database. In particular, there is good evidence that many “unknown unknowns” are actually “metabolites of metabolites” [150]. That is they are biotransformation products arising from phase I or phase II metabolism, from promiscuous enzyme reactions or from microbial transformations in the gut or from the environment. Ideally, if the chemical structures of these “novel” metabolites could be generated, then it may be possible to create a new library of theoretical but biologically reasonable metabolite structures. A number of commercial tools now exist for calculating phase I metabolism and phase I metabolite structures using known structures as the starting point [156]. Software is also starting to appear to predict promiscuous enzyme products [155] and microbial biotransformations [157] from known structures. Given the existence of these tools, it seems that the next logical step would be to bring them together and to start creating a large database of biologically reasonable or biologically feasible metabolites. Such a process may generate up to 5 million *never-before-seen* or *never-before-described* structures. Using this collection of theoretical metabolites and running it through the MS/MS and EI-MS prediction/interpretation tools described above could help with the identification of many “unknown-unknowns.” Preliminary efforts in this regard have recently been published and the results are quite promising [150]. One example from natural product research is a method that allowed the description of previously unreported alkaloids through the comparison of fragmentation patterns with the patterns of previously isolated ones and the MS/MS spectra simulation of in silico-predicted compounds [158].

While we have largely described theoretical approaches to solving the “unknown” metabolite problem, we expect that many of these ideas and software could (and should) be integrated into experimental metabolomics or natural product dereplication pipelines. Furthermore, by exploiting the power of NMR spectroscopy (for unambiguous compound ID) with the power of MS/MS and EI-MS (for approximate compound identification) it may be possible to create a much more efficient and far more accurate route to unknown compound identification [159]. No doubt other methods and other ideas for novel compound identification will emerge over the coming years. Likewise, many of the proposed techniques and many of the existing databases described here will continue to improve in their size, coverage, accuracy and robustness. Given the growing interest in this area and the growing realization that most of the metabolome that we now see is essentially unknown, it is likely that greater effort will be directed to unknown identification over the coming years. We are hopeful that within the next decade, routine measurement and identification of 70%–100% of any given metabolome, for any given biofluid from any given organism, will become possible.

## Figures and Tables

**Table 1:** List of common mass analyzers and instrument configurations detailing: mass resolution, approximate mass range, tandem MS/MS capabilities and acquisition speed. TOF = Time of Flight, TOF/TOF = Tandem TOF, IT = Ion Trap, FT-ICR = Fourier Transform Ion Cyclotron Resonance, Q-TOF = Quadrupole Time of Flight, Da = Dalton.

Mass Analyzer	Mass Resolution	Mass Range (Da)	MS/MS	MS^n^	Acquisition Speed
Quadrupole	~1000	50–6000	Yes	No	Medium
Ion Trap	~1000	50–4000	Yes	Yes	Medium
TOF	2500–40,000	20–500,000	No	No	Fast
TOF/TOF	>20,000	20–500,000	Yes	No	Fast
Orbitrap	>100,000	40–4000	Yes	Yes	Slow
FT-ICR	>200,000	10–10,000	Yes	Yes	Slow
Ion Mobility Q-TOF	13,000/40,000	Up to 40,000	Yes	No	Fast

**Table 2:** Basic principles for FTIR metabolite elucidation.

Does the FTIR spectra have a Carbonyl (C=O) band? Strong band at 1820–1660 cm^−1^	Yes	Acid	• Look for indications that an O–H band is present (broad absorption near 3300–2500 cm^−1^; will overlap the C–H stretch near 3000 cm^−1^).
			• Look for indications that a C–O single bond is present (1100–1300 cm^−1^).
			• Carbonyl band (near 1725–1700 cm^−1^).
		Ester	• Look for C–O absorption (medium intensity near 1300–1000 cm^−1^. There will be no O–H band (3600–3300 cm^−1^).
		Aldehyde	• Look for aldehyde e type C–H absorption bands (two weak absorptions to the right of the C–H stretch near 2850 cm^−1^ and 2750 cm^−1^).
			• Carbonyl band (near 1740–1720 cm^−1^).
		Ketone	• The weak aldehyde C–H absorption bands will be absent.
			• Carbonyl band (near 1725–1705 cm^−1^).
	No	Alcohol	• Look for OH band (broad adsorption at 3600–3300 cm^−1^).
			• Look for C–O absorption band (near 1300–1000 cm^−1^).
		Alkene	• Look for weak absorption near 1650 cm^−1^ for a double bond.
			• Look for CH stretch band near 3000 cm^−1^.
		Aromatic	• Look for the benzene double bonds (medium to strong absorptions near 1650–1450 cm^−1^).
			• The CH stretch band is much weaker than in alkenes.
		Alkane	• The main absorption will be the C–H stretch near 3000 cm^−1^.
			• Look for another band near 1450 cm^−1^.
		Alkyl bromide	• Look for the C–H stretch near 3000 cm^−1^.
			• Look for another band to the right of 667 cm^−1^.

## Abbreviations

The following abbreviations are used in this manuscript:

HSQC: Heteronuclear Single Quantum Coherence
COSY: Correlation spectroscopy
TOCSY: Total correlation spectroscopy
HMBC: Heteronuclear multiple-bond correlation spectroscopy
NOESY: Nuclear Overhauser effect spectroscopy
ROESY: Rotating frame nuclear Overhauser effect spectroscopy
